# The association between prenatal and early-life antibiotic use and obesity at 4–5 years in the Born in Bradford birth cohort

**DOI:** 10.1186/s12889-026-28420-y

**Published:** 2026-07-15

**Authors:** Lila Goodwin, Ellena Badrick, Gillian Santorelli, Sergio Souza da Cunha, Emily Petherick, Sam Oddie, Neil Pearce, John Wright, Lucy Pembrey

**Affiliations:** 1 London School of Hygiene & Tropical Medicine, London, UK; 2 Bradford Institute for Health Research, Bradford Teaching Hospitals NHS Foundation Trust, Bradford, UK; 3 School of Sport, Exercise and Health Sciences, Loughborough University, Loughborough, UK; 4 National Institute for Health Research (NIHR) Leicester Biomedical Research Centre, University Hospitals of Leicester NHS Trust and University of Leicester, Leicester, UK; 5 Department of Medical Statistics, London School of Hygiene & Tropical Medicine, Keppel Street, London, WC1E 7HT UK

**Keywords:** Antibiotic exposure, Pregnancy, Early-life, Childhood obesity, Born in Bradford, Birth cohort

## Abstract

**Background:**

The prevalence of childhood obesity has quadrupled globally over the last 30 years highlighting the urgency for robust research on modifiable risk factors. This study investigates the association between prenatal and early-life (0–24 months) antibiotic use and obesity at 4–5 years within the Born in Bradford (BiB) cohort, and whether this differs by ethnicity or by sex.

**Methods:**

BiB is a large, multi-ethnic birth cohort of 13 858 children in Bradford, UK. Data for children and their mothers were obtained from a baseline questionnaire, linked electronic health records (including maternity records and general practitioner prescriptions), and anthropometric measurements at school. Logistic regression models were employed to estimate the associations between antibiotic exposure in pregnancy and early-life and the outcome, having obesity at age 4–5 years, adjusting for several confounders.

**Results:**

In this population, nearly a tenth of children had obesity, just over a third were exposed to antibiotics in pregnancy, and nearly three quarters were exposed in the first two years of life. The adjusted analysis revealed no clear evidence of an association between prenatal antibiotic use and having obesity at 4–5 years (aOR 1.09; 95% CI 0.94, 1.27; *p* = 0.247). There was strong evidence of an association between early-life antibiotic use and having childhood obesity (aOR 1.36; 95% CI 1.14, 1.62; *p* = 0.001), and increasing numbers of prescriptions were associated with higher odds of obesity (aOR 1.058; 95% CI 1.030, 1.088; p_trend_ = 0.0001). No effect modification by ethnicity or sex was observed.

**Conclusion:**

These findings support those found in previous experimental animal and epidemiological studies, highlighting the importance of further investigation into early-life antibiotic exposure, infection burden, and later obesity risk.

**Supplementary Information:**

The online version contains supplementary material available at https://doi.org/10.1186/s12889-026-28420-y.

## Background

The proportion of children with obesity has been on the rise globally, quadrupling (from 2 to 8%) in the last 30 years [1]. In 2022/2023 in the UK, 9.2% of 4–5-year-olds and 22.7% of 10–11-year-olds were living with obesity, higher than the global average [2]. Obesity in childhood often persists into adulthood, doubling the risk of premature death [3]. Research on the modifiable risk factors for childhood obesity is important to design interventions that can reduce related morbidity and mortality.

The growth-promoting effects of antibiotics were first realised in the 1940 s, leading to their low-dose use in livestock farming [4, 5]. This was confirmed by experimental studies in animal models providing causal evidence for these observed effects [5, 6]. Earlier administration (in infancy) has been linked to more extreme weight gain both in agriculture [7] and in experimental studies in mice, with metabolic effects persisting into adulthood [6].

Antibiotic use is associated with changes in gut microbiota composition, favouring species that extract more energy from food, thus contributing to obesity [6–10]. Other mechanisms may modify or confound the antibiotic-obesity association. For example, the illness motivating the antibiotic prescriptions may result in parental behavioural changes that promote obesity, such as staying inside and not exercising [11].

Some previous studies found that prenatal antibiotic exposure was associated with an increased risk of having obesity. However, the current evidence is inconclusive, with several studies finding null associations and inconsistency in the magnitude of effect [12–16]. The largest effect size was reported by a prospective cohort study in Greece, where prenatal self-reported antibiotic use was associated with twice the odds of living with obesity at 6 years (risk ratio 2.09; 95% confidence intervals (CI) 1.58, 2.76) [14]. Only two previous studies investigated effect modification by sex [17, 18], but neither found evidence of this. Few studies have investigated differences by ethnicity, either because of predominantly White populations or small sample sizes.

There is strong evidence of an association between early-life (0–4 years) antibiotic use and childhood obesity, with several meta-analyses yielding positive associations [19–21]. The effect sizes (odds ratios) for individual studies ranged from 1.04–6.15 [12, 19–23]. The largest effect size was reported by a prospective cohort study in California, USA (adjusted odds ratio (aOR) 6.15; 95% CI 1.03, 36.70), although wide CI indicate some imprecision likely due to a small sample size (97 children) [23]. There is some evidence of a dose–response effect, with increasing number of prescriptions associated with greater odds of having childhood obesity [19]. Only one study reported the findings by ethnicity and found a weak overall association between antibiotic use and obesity (aOR 1.1; 95% CI 0.6, 1.8), but strong evidence of an association in Hispanic children (aOR 3.3; 95% CI 1.7, 6.4) [24]. Of the studies that investigated this association by sex, most found stronger associations in boys, suggesting potential sexual dimorphism, though the evidence for this is inconsistent [25–27].

While existing research has laid a foundation for understanding the association between antibiotic use and obesity, gaps remain, especially in understanding effect modification by ethnicity and sex. Several previous studies experienced problems associated with selection bias [14], small sample size [23] and small numbers of children with obesity [22]. This study addresses these limitations by investigating the association within the BiB cohort, a large multi-ethnic birth cohort. Children of Pakistani ethnicity have one of the highest prevalences of severe obesity in the UK [2], making research in this group a priority. To our knowledge, this study is the first to explore this association in children of Pakistani heritage.

## Methods

### Cohort information

BiB is a multi-ethnic prospective birth cohort established in 2007 in Bradford, West Yorkshire; nearly 50% of babies born there are of Asian/Asian British ethnicity (90% of whom are of Pakistani heritage). Bradford is one of the most deprived areas in England. Between September 2007 and December 2011, 12 453 women (13 776 pregnancies) were recruited from Bradford Royal Infirmary at 26–28 weeks’ gestation, resulting in 13 858 births, which are representative of all births in Bradford [28].

### Sources of data

Data on ethnicity, socio-economic factors and maternal behaviours during pregnancy were self-reported in a baseline questionnaire administered at recruitment. The population analysed was restricted to Asian/Asian British Pakistani and White British individuals. Ethnicity was defined as ethnic group (e.g. Asian/Asian British, White British) and cultural background (e.g. Pakistani, Bangladeshi, Indian). Socio-economic position (SEP) was defined using a BiB-specific latent class analysis of 19 component variables [29]. Early pregnancy/pre-pregnancy body mass index (BMI) was derived from height measured at pregnancy booking and weight measured at recruitment to BiB. Information on antibiotic prescriptions were extracted from linked electronic health records (supplementary material 1). Birth data was obtained from the hospital maternity records. Child BMI at 4–5 years was sourced from the National Child Measurement Programme in which school nurses measured the height and weight of children in reception (aged 4–5) using calibrated SECA scales and consistent operating procedures.

### Exposure definitions

Prenatal antibiotic exposure was categorised as a binary variable; children were considered as exposed if their mothers received any antibiotic prescriptions during pregnancy (see supplementary material 1 for prescription code list). This was further divided into 3 binary non-mutually exclusive variables corresponding to any prescriptions in each trimester: first trimester (< 93 days of gestational age); second trimester (93–184 days); third trimester (185 days of gestational age to 7 days before the child's birth). For the BiB cohort and in the UK generally, gestational age is estimated from the date of delivery and the 10-week ultrasound scan. Early-life antibiotic exposure was defined as both a binary variable of any antibiotic prescriptions between 0–24 months of age, and as a continuous variable of the total number of prescriptions during this period.

### Outcome definition

The outcome, having obesity at 4–5 years, was analysed as a binary variable (children with obesity/children not living with obesity), based on BMI z-scores. BMI was calculated from weight and height measurements taken by nurses using the following formula:

$$BMI= \frac{weight}{{height}^{2}}$$

Standardised BMI z-scores were calculated for each child’s BMI using the LMS method (LMS growth, Health for all children) based on the UK90 reference data [30, 31], which is recommended for children aged 4 and over and is used by the National Child Measurement Programme [2]. Children were defined as living with obesity if they had a BMI z-score above the 95th percentile [32].

### Covariate definitions

For mother’s BMI, an adjustment was applied to measurements taken at > 13 weeks’ gestation (2 580 mothers), as women begin to experience gestational weight gain at that time [33]. This adjustment was based on an average weight gain of 12.48 kg between weeks 14–40 of gestation [33] as found in previous studies, assuming a linear relationship between week of gestation and weight gain (0.48 kg per week). The same adjustment was applied to all women due to a lack of research on trajectories by ethnicity:

$${BMI}_{\mathrm{pre-pregnancy}}={BMI}_{\mathrm{pregnancy}}-\frac{(GA-13)\times0.48}{\mathrm{height}^2}$$

This adjustment resulted in a reduction in mean BMI for the 2 580 women who had their BMI measured past 13 weeks from 26.35 (SD 5.61) to 25.39 (SD 5.56), reflecting a more accurate pre-pregnancy BMI. Pre-pregnancy BMI was categorised as a binary variable (mothers with pre-pregnancy obesity/mothers without pre-pregnancy obesity). Mothers were defined as living with obesity if they had a pre-pregnancy BMI of ≥ 30 kg/m2 [34] for White British women, and BMI of ≥ 27.5 kg/m2 for Pakistani women, based on updated cut-offs for Asian (including Pakistani) individuals [35].

### Statistical analysis

All analyses were performed using Stata SE 18.0.

**Fig. 1:**
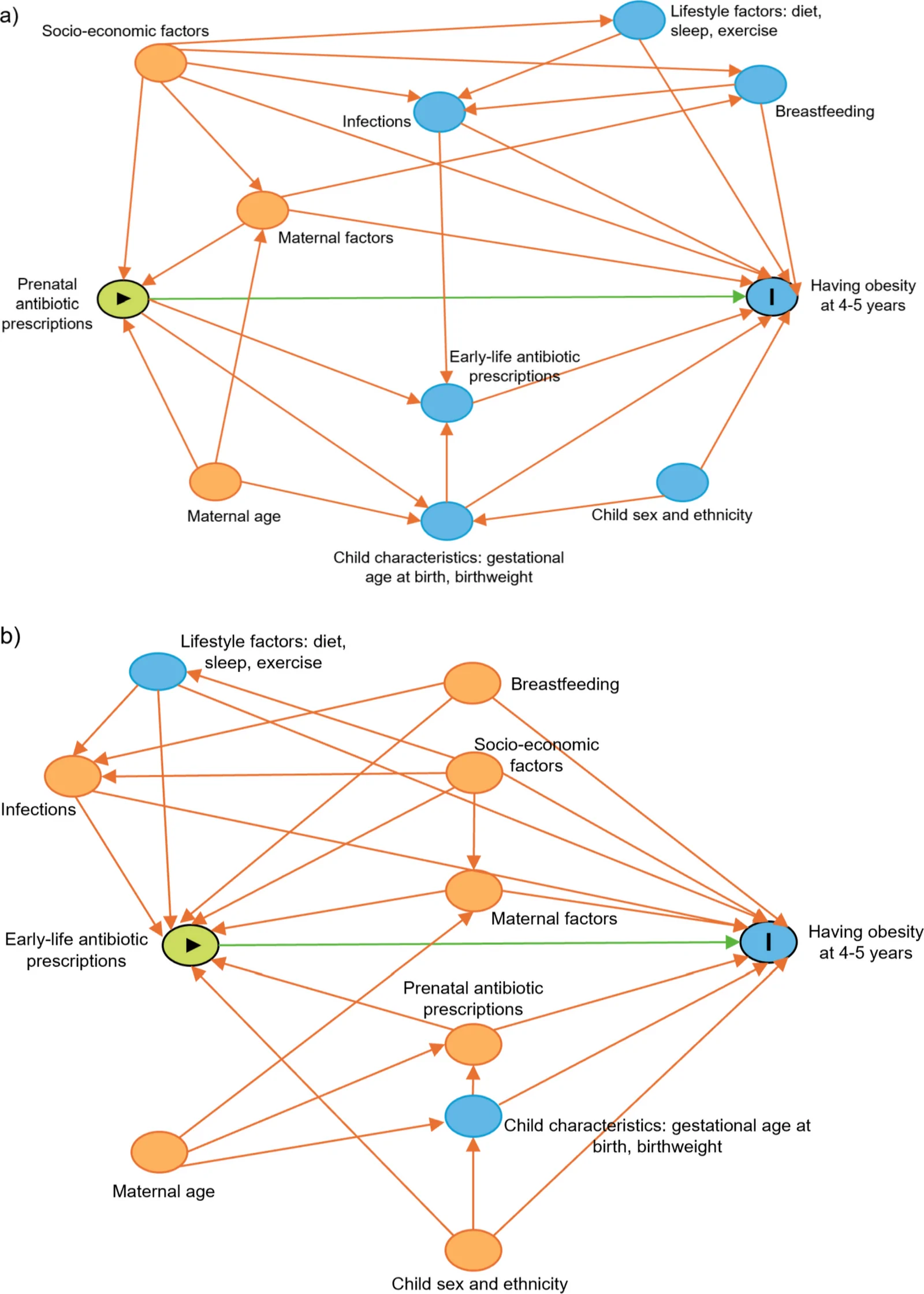
Directed acyclic graphs for the association between (**a**) prenatal and (**b**) early-life antibiotic use and childhood obesity, based on previous research. Designed in Dagitty [37]. Green with triangle = exposure, blue with I = outcome, orange = potential confounders, blue = other variables. Note maternal factors includes smoking during pregnancy, gestational diabetes, pre-pregnancy obesity and parity

The study population’s characteristics were summarised, stratified by obesity status and ethnicity. Baseline characteristics of those missing obesity and antibiotic data were compared to those without missing data to assess selection bias. Sex (assigned at birth and classified as male or female) and ethnicity were included a priori in all models. Potential confounding variables were identified a priori using directed acyclic graphs informed by existing literature and subject-matter knowledge (Fig. 1) [36, 37]. Potential confounders were considered for inclusion in the regression models if they were (1) a hypothesised risk factor for obesity, (2) hypothesised to be independently associated with prenatal and/or early-life antibiotic exposure and (3) not on the causal pathway. Descriptive cross-tabulations and univariable analyses were used to examine covariate distributions and relationships with exposure and outcome, but not to determine the final adjustment set. Confounders were investigated separately for prenatal and early-life exposure. Unadjusted analyses with each exposure, outcome, sex and ethnicity were conducted to obtain crude odds ratios (OR), 95% CI and Wald test p-values. Fully adjusted models included the a priori confounders identified for each exposure.

All variables identified in the a priori criteria for prenatal or early-life exposure were included in their respective logistic regression models. Standard errors from each fully adjusted model were compared with those from unadjusted models to assess collinearity. If evidence of collinearity had been observed pseudo-R^2^ would have been calculated to assess the extent of the collinearity and the contribution of the variable to model fit [36]. However, no evidence of collinearity was observed and no variables were removed on this basis.

All siblings were included in the analyses to maximise power and generalisability, as there is evidence that risk of obesity varies with parity [38]. Robust standard errors clustered at the maternal level were used to account for non-independence among siblings born to the same mother.

### Sensitivity analysis

A sensitivity analysis with only first siblings (*N* = 9 371) was conducted to confirm results with truly independent observations. Some studies have shown that obesity estimated by body fat percentage was similar to overweight/obesity estimated by BMI [39]. Overweight is also the precursor to obesity and is associated with adverse health outcomes [1]. Therefore, a second sensitivity analysis included children with overweight (z-scores *≥* 85th percentile) in the outcome. To explore differences across the BMI spectrum, we conducted a sensitivity analysis using a four-level outcome: underweight (≤ 2nd percentile), healthy weight (> 2nd to < 85th), overweight (≥ 85th to < 95th), and obesity (≥ 95th) [32]. Previous studies have shown that breastfeeding could confound the association between antibiotic use and having obesity [22, 27, 40]. Breastfeeding data were not available for all children so a subgroup analysis (*N* = 8 991) was conducted to investigate the effect of breastfeeding in this population (supplementary material 2).

### Ethical approval

The original BiB study received ethical approval from the Bradford Research Ethics Committee (Ref 07/H1302/112). Ethical approval for this study was granted by the London School of Hygiene and Tropical Medicine ethics committee (Ref 30,315).

## Results

### Selection of study population

**Fig. 2:**
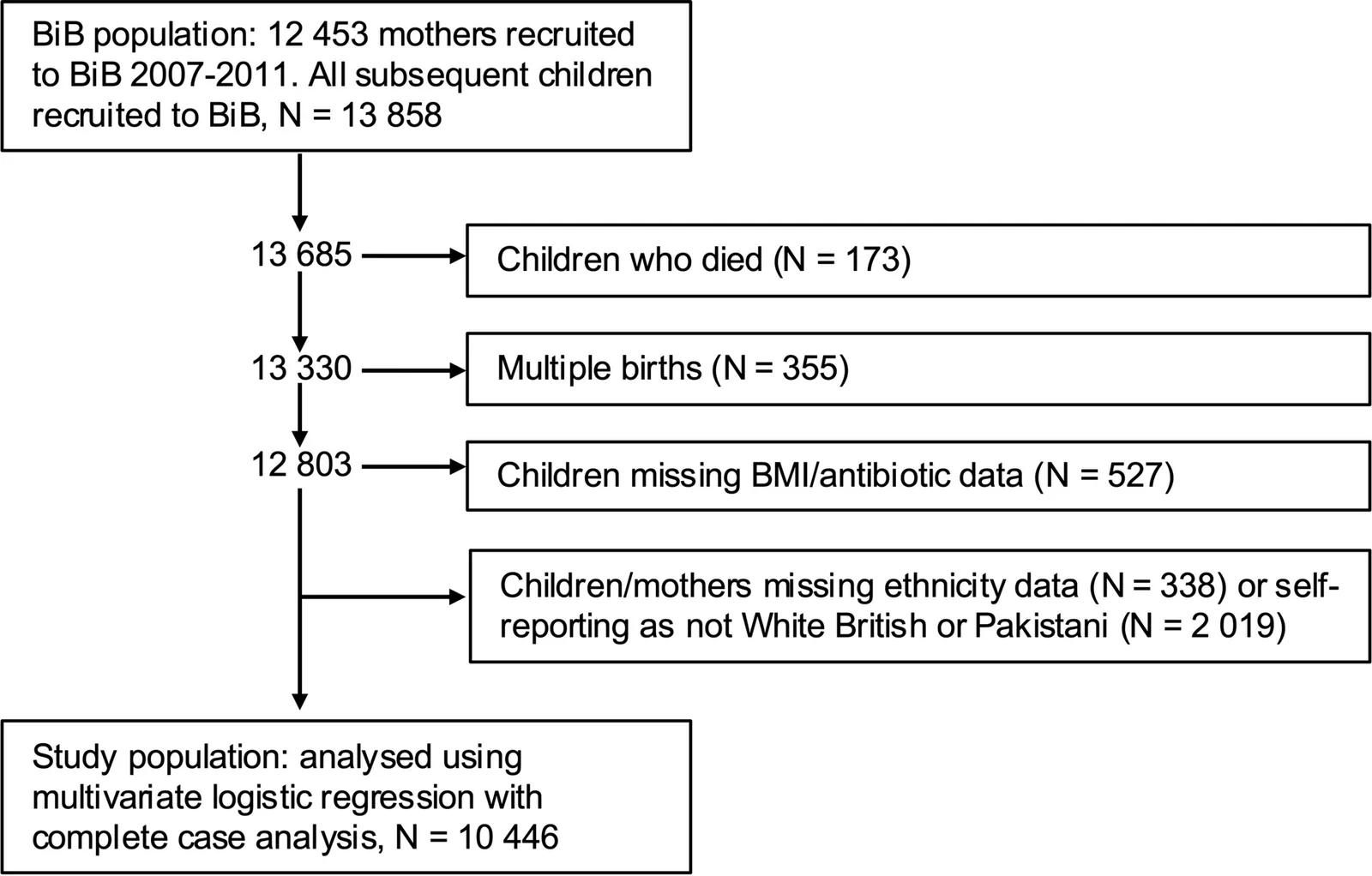
The selection process of the study population

The study population included all singleton live births in the BiB cohort that had complete BMI, antibiotic exposure, and ethnicity data and were of Pakistani or White British ethnicity (*N* = 10 446) (Fig. 2).

### Baseline characteristics

Nearly half of the population were in the most socioeconomically disadvantaged groups. In this study, 72% of children were exposed to antibiotics in their first 2 years of life while 36% were exposed prenatally (Table 1). Nearly a tenth of children were classified as having obesity (*N* = 970) with a further 1 117 (11%) classified as having overweight. Having obesity was more common in those prenatally exposed to antibiotics (10%) and those exposed in early-life (10%), than those who were unexposed (9% and 8%, respectively) (Table 1). Obesity was more prevalent in children of mothers who smoked during pregnancy (11%), had gestational diabetes (15%), or had pre-pregnancy obesity (14%) than in the overall study population (9%). Obesity was also more common in the most deprived SEP group (10%) than in the least deprived group (8%) (Table 1). The study population is broadly representative of the larger BiB cohort (supplementary material 3).

**Table 1:** Baseline characteristics of the study population (*N* = 10 446) with stratification by obesity at 4–5 years; OR and 95% CI quantifying the association between each variable and obesity are from univariate logistic regression models and *p*-values from likelihood ratio tests

			Total (*N* = 10 446), n(column%)	Missing	Have obesity (*N* = 970), n(row%)	Do not have obesity (*N* = 9 476), n	OR (95% CI)	*p*-value
Child characteristics								
Body mass index		Healthy/underweight	8 359 (80.02)					
		Overweight	1 117 (10.69)	_	_	_	_	_
		Obese	970 (9.29)					
Prenatal Antibiotic Exposure	1 st trimester	No	8 730		803 (9.20)	7 927	1	
		Yes	1 716 (16.43)		167 (9.73)	1 549	1.064 (0.893, 1.268)	0.489
	2nd trimester	No	8 694		778 (8.95)	7 916	1	
		Yes	1 752 (16.77)	_	192 (10.96)	1 560	1.252 (1.060, 1.480)	0.01
	3rd trimester	No	8 955		814 (9.09)	8 141	1	
		Yes	1 491 (14.27)		156 (10.46)	1 335	1.169 (0.975, 1.400)	0.096
	Any trimester	No	6 681		588 (8.80)	6 093	1	
		Yes	3 765 (36.04)		382 (10.15)	3 383	1.170 (1.022, 1.340)	0.024
Early-life antibiotic exposure (0–24 months)		No	2 883	_	220 (7.63)	2 663	1	
		Yes	7 563 (72.40)		750 (9.92)	6 813	1.333 (1.139, 1.559)	0.0002
Sex		Female	5 072		467 (9.21)	4 605	1	
		Male	5 374 (51.45)	0	503 (9.36)	4 871	1.018 (0.892, 1.162)	0.788
Ethnicity		White British	4 739	_	446 (9.41)	4 293	1	
		Pakistani	5 707 (54.63)		524 (9.18)	5 183	0.973 (0.852, 1.111)	0.687
Gestational age		Normal term	9 746		907 (9.31)	8 839	1	
		Preterm (< 37 weeks)	542 (5.27)	158	53 (9.78)	489	1.056 (0.789, 1.414)	0.715
Birthweight		Healthy	9 538		907 (9.51)	8 631	1	
		Low (< 2500 g)	749 (7.28)	159	53 (7.08)	696	0.725 (0.544, 0.966)	0.022
Mode of delivery		Vaginal	8 044		736 (9.15)	7 308	1	
		Caesarean	2 222 (21.64)	180	222 (9.99)	2 000	1.102 (0.941, 1.291)	0.231
Maternal characteristics								
Smoking during pregnancy		No	9 245		841 (9.10)	8 404	1	
		Yes	1 200 (11.49)	1	129 (10.75)	1 071	1.204 (0.989, 1.464)	0.069
Consumed Alcohol in pregnancy or 3 months before		Yes	2 870 (32.00)		268 (9.34)	2 602	1	
		No	6 098		573 (9.40)	5 525	1.007 (0.865, 1.173)	0.929
		Don’t remember	< 10	1 473	0	< 10		
Gestational Diabetes		No	10 313		953 (9.24)	9 360	1	
		Yes	114 (1.09)	19	17 (14.91)	97	1.721 (1.023, 2.894)	0.041
Country of birth		Non-UK	2 837 (31.52)		259 (9.13)	2 578	1	0.599
		UK	6 163	1 446	584 (9.48)	5 579	1.042 (0.894, 1.215)	
Employed at baseline		Yes	3 813 (42.41)		339 (8.89)	3 474	1	
		No	5 178	1 455	503 (9.73)	4 674	1.105 (0.956, 1.277)	0.175
Education Higher than A-level		No	6 335		612 (9.66)	5 723	1	
		Yes	2 031 (24.28)	2 080	169 (8.32)	1 862	0.849 (0.710, 1.014)	0.068
Parity at child’s birth		Primiparous	3 797 (38.30)		361 (9.51)	3 436	1	
		Multiparous	6 118	531	561 (9.17)	5 557	0.961 (0.836, 1.104)	0.574
Socio-economic position^a^		1 (Least deprived)	1 537 (17.15)		120 (7.81)	1 417	1	
		2	1 845 (20.59)		166 (9.00)	1 679	1.167 (0.914, 1.492)	
		3	1 336 (14.91)		137 (10.25)	1 199	1.349 (1.044, 1.744)	
		4	2 799 (31.23)		272 (9.72)	2 527	1.271 (1.016, 1.591)	
		5 (Most deprived)	1 445 (16.12)	1 484	145 (10.03)	1 300	1.317 (1.022, 1.697)	0.121
Age (years)		15–24	3 481 (33.32)		326 (9.37)	3 155	1	0.512
		25–34	5 676 (54.34)		514 (9.06)	5 162	0.964 (0.833, 1.115)	
		35–49	1 289 (12.34)	0	130 (10.09)	1 159	1.086 (0.876, 1.345)	
Pre-pregnancy Body mass index		Do not have obesity	6 340		464 (7.32)	5 876	1	
		Have obesity	2 641 (29.41)	1 465	379 (14.35)	2 262	2.122 (1.837, 2.451)	<0.0001

*Abbreviations*: *N* sample size, *n* number experiencing variable, *OR* Odds ratio, *CI* Confidence intervals

^a^Socio-economic position (SEP) was defined using a BiB specific latent class analysis of 19 component variables [29]

Over half of children were of Pakistani ethnicity. A fifth of White British women smoked during pregnancy and 68% consumed alcohol during pregnancy or three months prior, compared to only 3% of Pakistani women who smoked and less than 1% who drank alcohol. Gestational diabetes was twice as prevalent in Pakistani women compared to White British. Pre-pregnancy obesity was also more common in Pakistani mothers (32%) compared to White British (26%). For further baseline characteristics stratified by ethnicity see supplementary material 3.

### Univariable analysis

Confounding variables were determined to be:

For prenatal antibiotic exposure: gestational diabetes, SEP, smoking during pregnancy, maternal pre-pregnancy obesity. For early-life antibiotic exposure: low birthweight, SEP, smoking during pregnancy, maternal pre-pregnancy obesity, prenatal antibiotic use.

### Multivariable analysis

#### Prenatal antibiotic exposure

In the unadjusted logistic regression model, prenatal antibiotic exposure was associated with 1.15 times the odds of obesity (95% CI 1.00, 1.33) (Table 2). After adjusting for all additional confounders there was no evidence of an overall association (aOR 1.09; 95% CI 0.94, 1.27; *p* = 0.247).

**Table 2:** Association between prenatal antibiotic exposure and obesity at 4–5 years (*N* = 10 446); in any trimester and by trimester, and any trimester stratified by ethnicity (Pakistani and White British) and sex

	Crude OR* (95% CI), *p*-value	aOR** (95% CI), *p*-value	*p*-value^i^
Any trimester	1.152 (0.996, 1.332), 0.057	1.091 (0.941, 1.265), 0.247	_
By trimester			
First trimester	0.952 (0.784, 1.157), 0.625	0.925 (0.759, 1.127), 0.439	_
Second trimester	1.223 (1.019, 1.469), 0.031	1.171 (0.973, 1.408), 0.095	_
Third trimester	1.110 (0.890, 1.348), 0.349	1.051 (0.856, 1.290), 0.634	_
Exposure in any trimester stratified by ethnicity			
White British	1.186 (0.959, 1.466), 0.115	1.103 (0.889, 1.369), 0.373	0.892
Pakistani	1.112 (0.919, 1.369), 0.259	1.081 (0.883, 1.322), 0.450	
Exposure in any trimester stratified by sex			
Female	1.017 (0.824, 1.256), 0.874	0.961 (0.777, 1.189), 0.716	0.105
Male	1.290 (1.055, 1.577), 0.013	1.223 (1.000, 1.503), 0.050	

OR, aOR, 95% CI and p-values are from logistic regression models with robust standard errors

*Abbreviations*: *N* sample size, *OR* Odds ratio, *aOR* adjusted odds ratio, *CI* Confidence intervals

^i^from Wald tests for interaction with cluster-robust standard errors

^*^Adjusted for a priori confounders, sex and ethnicity

^**^Additionally adjusted for diabetes, maternal pre-pregnancy obesity, socio-economic position, smoking during pregnancy

When analysed by ethnicity, there was no evidence of an association in either White British (aOR 1.10, 95% 0.89, 1.37; *p* = 0.373) or Pakistani children (aOR 1.08, 95% CI 0.88, 1.32; *p* = 0.450) (Table 2), and no evidence for effect modification (*p* = 0.892). Stratification by sex showed a larger point estimate in male children (aOR 1.22, 95% CI 1.00, 1.50; *p* = 0.050), whereas the point estimate was closer to the null in females (aOR 0.96, 95% CI 0.77, 1.19; *p* = 0.716) (Table 2). However, a formal Wald test did not provide strong evidence of effect modification by sex (*p* = 0.105).

There was evidence of an unadjusted association between antibiotic exposure in the second trimester and obesity at 4–5 years (OR 1.22 (95% CI 1.02, 1.47), but not the first (OR 0.95; 95% CI 0.78, 1.16) or third trimester (OR 1.11; 95% CI 0.89, 1.35) (Table 2). After adjustment, the OR for all trimesters decreased, and there was weak evidence (*p* = 0.095) for an association between second trimester antibiotic exposure and obesity (aOR 1.17; 95% CI 0.97, 1.41) (Table 2).

#### Early-life antibiotic exposure

There was strong evidence of an unadjusted (OR 1.38; 95% CI 1.16, 1.63; *p* < 0.001) and adjusted (aOR 1.36; 95% CI 1.14, 1.62; *p* = 0.001) (Table 3) association between early-life antibiotic use and obesity.

**Table 3:** Association between early-life antibiotic exposure and obesity at 4–5 years (*N* = 10 446), stratified by ethnicity (Pakistani and White British) and sex

	Crude OR* (95% CI), *p*-value	aOR** (95% CI), *p*-value	*p*-value^i^
All	1.378 (1.161, 1.634), < 0.001	1.362 (1.144, 1.621), 0.001	_
Stratified by ethnicity			
White British	1.318 (1.047, 1.660), 0.019	1.342 (1.062,1.696), 0.014	0.828
Pakistani	1.450 (1.124, 1.841), 0.004	1.385 (1.070, 1.792), 0.013	
Stratified by sex			
Female	1.575 (1.233, 2.012), < 0.001	1.541 (1.202, 1.974), 0.001	0.147
Male	1.201 (0.949, 1.520), 0.128	1.199 (0.944, 1.525), 0.137	

OR, aOR, 95% CI and p-values are from logistic regression models with robust standard errors

*Abbreviations*: *N* sample size, *OR* Odds ratio, *aOR* adjusted odds ratio, *CI* Confidence intervals

^i^from Wald tests for interaction with cluster-robust standard errors

^*^Adjusted for a priori confounders sex and ethnicity

^**^Additionally adjusted for maternal pre-pregnancy obesity, birthweight, socio-economic position, smoking during pregnancy, prenatal antibiotic use

Stratification of the adjusted model by ethnicity showed strong evidence for an association in both Pakistani children (aOR 1.39, 95% CI 1.07, 1.79; *p* = 0.013) and White British children (aOR 1.34, 95% CI 1.06, 1.70; *p* = 0.014) (Table 3). A formal test for interaction presented no evidence for effect modification by ethnicity (*p* = 0.828). Stratification by sex showed a larger point estimate in females (aOR 1.54; 95% CI 1.20, 1.97; *p* = 0.001) than males (aOR 1.20; 95% CI 0.94, 1.53; *p* = 0.137) (Table 3), although a formal test for interaction provided no evidence for effect modification by sex (*p* = 0.147).

Children receiving *≥* 10 antibiotic prescriptions between 0–24 months of age had the highest proportion of obesity (Table 4). There was strong evidence of a dose–response relationship between the number of early-life antibiotic prescriptions and odds of obesity after adjusting for confounders (p-value for linear trend = 0.0001). Each additional prescription was associated with a 5.8% increase in odds of obesity (95% CI 3.0%, 8.8%) (Table 4). When examined as a categorical variable, every number of antibiotic prescriptions (except 8) was associated with increased odds of obesity, in comparison with those without antibiotics, although sample size and precision decreased with increasing numbers of prescriptions. There was no evidence to suggest a more complex (non-linear) association (p-value for departures from linear trend = 0.295) (Table 4).

**Table 4:** Association between number of antibiotic prescriptions (between 0–24 months of age) and obesity at age 4–5, *N* = 10 446

		Children with obesity n/N (%)	Model 4a: continuous			Model 4b: categorical		
			Crude OR* (95% CI)	aOR** (95% CI)	*p*-value^a^	Crude OR* (95% CI)	aOR** (95% CI)	*p*-value^b^
Number of antibiotic prescriptions	0	220/2 883 (7.63)	1			1		
	1	201/2 339 (8.59)	1.068 (1.040, 1.097)	1.058 (1.030, 1.088)	0.0001	1.194 (0.964, 1.481)	1.201 (0.966, 1.493)	0.295
	2	166/1 655 (10.03)				1.360 (1.081, 1.713)	1.380 (1.093, 1.742)	
	3	119/1 192 (9.98)				1.402 (1.089, 1.806)	1.412 (1.092, 1.826)	
	4	67/709 (9.45)				1.386 (1.022, 1.879)	1.371 (1.008, 1.864)	
	5	59/509 (11.59)				1.671 (1.205, 2.319)	1.539 (1.097, 2.159)	
	6	36/336 (10.71)				1.385 (0.918, 2.090)	1.383 (0.912, 2.100)	
	7	30/260 (11.54)				1.782 (1.164, 2.729)	1.645 (1.065, 2.540)	
	8	12/164 (7.32)				0.907 (0.469,1.755)	0.804 (0.397, 1.630)	
	9	16/118 (13.56)				2.024 (1.103, 3.714)	1.882 (1.029, 3.443)	
	>=10	44/281 (15.66)				2.300 (1.574, 3.361)	2.142 (1.444, 3.178)	

OR, aOR and 95% CI are from logistic regression models with robust standard errors

*Abbreviations*: *n* number of children with obesity, *N* total, *OR* Odds ratio, *aOR* adjusted odds ratio, *CI* Confidence intervals, *H0* null hypothesis, *H1* alternate hypothesis

^a^p-value from Wald test testing H0 of no association against H1 of a linear association (model 4a)

^b^p-value from Likelihood ratio test testing H0 of a linear association (model 4a) against H1 of a more complex association (model 4b) (without robust standard errors)

^*^Adjusted for a priori confounders sex and ethnicity

^**^Additionally adjusted for maternal pre-pregnancy obesity, birthweight, socio-economic position, smoking during pregnancy, prenatal antibiotic use

### Sensitivity analysis

See supplementary material 5 for the sensitivity analysis result tables. The sensitivity analysis including only first siblings confirmed the main analysis findings for prenatal and early-life exposure.

With obesity and overweight as the outcome (sensitivity analysis 2 in supplement material) there was weak evidence of an association with prenatal antibiotic exposure with narrower 95% CI than the main analysis (aOR 1.11; 95% CI 1.00, 1.24). There was some evidence for effect modification by ethnicity (*p* = 0.020) with evidence of an association in Pakistani children (aOR 1.26; 95% CI 1.08, 1.47; *p* = 0.003) but not White British children (aOR 0.98; 95% CI 0.84, 1.14; *p* = 0.755). Findings by sex and by trimester were consistent with the main analysis. The results also supported the main findings for early-life antibiotic exposure.

In the sensitivity analysis using categorical BMI (four levels), results were consistent with the main analysis. There was no evidence of association between prenatal antibiotic exposure and underweight, overweight, or obesity versus healthy weight. Early-life antibiotic exposure was associated with higher relative risk of obesity versus healthy weight (aRRR 1.388; 95% CI 1.165, 1.653; p < 0.001).

In the subgroup analysis (*N* = 8 991) with breastfeeding data, additional adjustment for breastfeeding did not materially alter the association between early-life antibiotic exposure and obesity (supplementary material 2).

## Discussion

### Summary of findings

Overall, there was no clear evidence for an association between prenatal antibiotic exposure and obesity at 4–5 years. There was no evidence for effect modification by sex or ethnicity.

Children receiving at least one antibiotic prescription between 0–24 months of age had 1.36 times the odds of obesity at 4–5 years compared to children who did not receive any prescriptions (*p* = 0.001). There was no evidence of effect modification by sex or ethnicity. Early-life prescriptions had a dose–response effect on obesity, with each additional antibiotic prescription being associated with a 5.8% increase in odds of having obesity.

### Interpretation

Obesity prevalence in this cohort is 9.3%, in line with the national average for 2015 of 9.1% [41]. The results support previous studies in the USA, Japan and China [16–18], finding no evidence of an association between antibiotic use in pregnancy and having obesity in childhood. Three studies that found associations between antibiotic use in pregnancy and obesity measured obesity at age 7 or later [16] indicating there may be longer term effects of antibiotic use on obesity not captured in our study. We observed the strongest association with antibiotic exposure in the second trimester. This has been found by previous studies, including a large cross-sectional study in New Zealand [12] and a prospective cohort study in California [15]. The tunica mucosa layer of the gut forms during the second trimester [42] which could influence gut microbiome vulnerability however this assumes gut colonisation begins in utero despite evidence for the sterile womb hypothesis [43].

Our study, in line with several systematic reviews and an observational study in the UK [19–21], found strong associations between early-life antibiotic use and obesity. One previous study that did not find associations used BMI self-reported from parents [19]. Parental self-reported BMI has been shown to underestimate the weight of heavier children and overestimate the weight of lighter children [44], which could underestimate the true association.

A causal interpretation is biologically plausible, but this observational analysis cannot distinguish the effects of antibiotic exposure from underlying infection burden, healthcare-seeking behaviour, or related immune and inflammatory pathways. One potential mechanism for the association between antibiotic use and childhood obesity is via the gut microbiome. Experimental animal studies have shown that a faecal microbiota transplant from an adult female human with obesity to germ-free mice induced an obese phenotype in the mice [45]. Observational human and experimental animal studies have shown that antibiotic use alters gut microbiome composition, reducing abundance of several species considered protective against obesity [6] and increasing abundance of species associated with having obesity, such as *Bifidobacterium* [9, 10]. The sensitivity analysis using a four-level BMI outcome suggests that antibiotic use is primarily associated with obesity rather than a graded increase across BMI categories. One possible explanation is that antibiotic-induced disruption of the gut microbiome increases susceptibility to obesity, potentially interacting with other obesity-related factors such as diet. Some studies suggest gut microbiome establishment begins in utero [46], however this study suggests it is most susceptible to the effects of antibiotics in the first two years of life, likely due to direct exposure rather than prenatal exposure though the mother.

Early-life antibiotic use could also be a proxy for the underlying infection for which they were prescribed. Previous studies found infection is bi-directionally associated with obesity, mediated by inflammation [47], which could partially explain this observed association. In addition, infection may mediate the observed association, as antibiotic use may influence subsequent infection risk through effects on the microbiome [48]. These pathways could not be separated in this analysis.

In this study the association between prenatal antibiotic exposure and having obesity had a larger point estimate in males than females. However, there was no evidence of effect modification by sex (*p* = 0.105). Previous studies in Japan and China, that measured obesity at 1–4 years, found higher effect sizes for females [17, 18]. These differences may reflect variation between countries/populations or be due to chance.

In the association between early-life antibiotic use and obesity, stratification by sex showed a larger point estimate in females, although there was no evidence of effect modification. Most previous studies that investigated effect modification by sex, found stronger associations in males, although none adjusted for prenatal antibiotic exposure, which could partially explain this difference.

To our knowledge, this study was the first to investigate this association in children of Pakistani heritage, and ethnicity did not modify the association despite children aged 4–5 years of Pakistani heritage being at increased risk of severe obesity in the UK [2, 41].

### Strengths

This study has several strengths. Its large sample size provides sufficient power to detect effect modification by sex and ethnicity; flexible recruitment strategies, with multi-lingual staff, minimised selection bias; nurses measuring anthropometric measurements were blinded to children’s antibiotic exposure status; low proportion of missing data for antibiotic exposure and obesity (< 5%); establishment of temporality as exposure was measured prior to outcome; and high-precision equipment, such as SECA scales, were used for measurement.

### Limitations

In the UK, up to 50% of prescribed medications, including antibiotics, are not taken as directed [49], which can lead to exposure misclassification. This would lead to false positives if people do not take their prescriptions, resulting in bias toward the null. Few people (1%) report access to antibiotics without prescription [50] making false negatives less likely.

Outcome misclassification may occur due to BMI’s low sensitivity (14.3%−60%) [51], underestimating obesity compared to body fat percentage or skinfold thickness [39, 51]. However, other studies found obesity prevalence detected by body fat percentage was almost identical to BMI z-score > 85th percentile [39]. The latter was used to define the outcome in the sensitivity analysis. Unmeasured residual confounding could partially explain the antibiotic-obesity association. The low pseudo R^2^ suggests that a large portion of the variability in obesity remains unexplained by the model, possibly due to unmeasured factors like exercise, diet, sleep and infections (Fig. 1). While UK growth charts (UK90) were used for both British Pakistani and White British children to allow for direct ethnic comparisons, it is important to acknowledge that growth trajectory differences by ethnicity have been observed [52]. Excluding multiple births limits generalisability to singleton births.

Antibiotic exposure was higher in the study population, (especially in early-life (72%)) than expected UK-wide [53]. Previous BiB studies suggest that higher prescribing in this setting reflects greater underlying health need associated with deprivation and early-life infection burden, rather than overprescribing [54]. Therefore, generalisability is strongest for cities with similar deprivation, early-life infection burden and prescribing practices.

### Recommendations for research

Future work should investigate the impact of specific antibiotic types, dosage and adherence to prescribed antibiotic regimens. Several studies found self-reported antibiotic use underestimates actual prescriptions or pharmacy records [55], however, it is unclear whether this is discrepancy is due to recall bias or non-adherence. Therefore, a combination of methods is recommended to accurately capture antibiotic exposure. Future research should aim to disentangle the extent to which antibiotic exposure reflects infection burden, healthcare-seeking behaviour, and potential biological pathways linking immune development and later obesity. To address concerns about the sensitivity of BMI, future research should compare different BMI z-score thresholds with a gold standard like dual-energy X-ray absorptiometry [56]. A previous BiB study found that South Asian children had a greater mean total body fat percentage than White children within the same BMI category [57]. This reinforced the need for ethnicity-specific BMI thresholds to improve sensitivity, while maintaining high specificity in obesity classification, across different ethnicities. Future studies should assess whether these associations persist in later childhood, beyond adiposity rebound, and in other populations within and outside the UK.

## Conclusion

This study provides observational evidence of the association between early-life antibiotic use and increased obesity at 4–5 years in a multi-ethnic, socioeconomically deprived UK city, consistent with previous animal and human studies. No evidence of effect modification by sex or ethnicity (British Pakistani and White British) was found, despite the higher risk of obesity-related morbidity in individuals of South Asian ethnicity observed in previous studies [2, 58]. The diverse demographics of the BiB cohort provided the statistical power to detect these associations by ethnicity, overcoming limitations of previous studies.

The findings are relevant to other UK cities with similar demographic profiles, particularly those with high early-life infection burden, deprivation [59] and South Asian populations. Further investigation is required to validate these findings, disentangle the effects of antibiotic use from infection, and explore whether this association persists into adolescence and adulthood. The findings highlight the potential unintended long-term consequences of antibiotic use, reinforcing the importance of appropriate antibiotic stewardship.

## Supplementary Information

Supplementary Material 1.

## Data Availability

The datasets generated during and/or analysed during the current study are not publicly available due to ethical restrictions. However, researchers are encouraged to make use of the BiB data, which are available through a system of managed open access via the Born in Bradford research team. Please see our online guidance (https://borninbradford.nhs.uk/research/guidance-for-collaborators/).

## Abbreviations

BiB: Born in Bradford
BMI: Body mass index
CI: Confidence intervals
OR: Odds ratio
aOR: Adjusted odds ratio
SEP: Socio-economic position
LRT: Likelihood ratio test
